# Development of an alcohol-inducible gene expression system for recombinant protein expression in *Chlamydomonas reinhardtii*

**DOI:** 10.1007/s10811-018-1480-8

**Published:** 2018-04-24

**Authors:** Sujin Lee, Yong Jae Lee, Saehae Choi, Su-Bin Park, Quynh-Giao Tran, Jina Heo, Hee-Sik Kim

**Affiliations:** 1 Cell Factory Research Center, Korea Research Institute of Bioscience and Biotechnology (KRIBB), Daejeon, 34141 Republic of Korea; 2 Department of Environmental Biotechnology, KRIBB school of Biotechnology, Korea University of Science and Technology (UST), Daejeon, 34113 Republic of Korea; 3 Present Address: K-Biohealth, Osong, Chungbuk, 28160 Republic of Korea

**Keywords:** Alcohol-inducible promoter, Gene expression, Microalgae, *Chlamydomonas reinhardtii*

## Abstract

Microalgae have been widely considered for the production of valuable products, such as lipid-based biofuel, value-added pigments, and anti-photo aging reagents. More recently, microalgae have been considered an alternative host for recombinant protein production because of their economic benefits and ecofriendly characteristics. Additionally, many microalgal strains identified to date are generally recognized as safe (GRAS); therefore, the use of microalgae-based technology is promising. However, basic studies on the genetic engineering of microalgae are rare, despite their importance. Particularly, inducible promoter systems that can be applied for strain engineering or recombinant protein production are rarely studied; hence, a number of challenging issues remain unsolved. Therefore, in this study, we focused on the development of a convenient and compact-inducible promoter system that can be used in microalgae. Based on previous success with plant systems, we employed the alcohol-inducible AlcR-P_*alc*A_ system, which originates from the filamentous fungus, *Aspergillus nidulans*. This system comprises only two components, a regulatory protein, AlcR, and an inducible promoter, P_*alc*A_. Therefore, construction and transformation of the gene cassettes can be easily performed. Ethanol-dependent gene expression was observed in the transformants with no significant growth retardation or inducer consumption observed in the cells cultivated under optimized conditions.

**Electronic supplementary material:**

The online version of this article (10.1007/s10811-018-1480-8) contains supplementary material, which is available to authorized users.

## Introduction

Global warming, caused by increased concentrations of greenhouse gases, is one of the most important environmental issues of the current era and is hence the focus of attention. In addition, carbon capture, utilization, and storage strategies are generating interest to address the issues associated with global warming (Demirbas 2011). Microalgae are unicellular photosynthetic microorganisms, typically found in both freshwater and marine systems. These organisms have great potential for bioremediation, particularly for the flue gases encompassing carbon dioxide (CO_2_), nitrogen oxides (NO_X_), and sulfur oxides (SO_X_) (Yen et al. 2015). In addition, many microalgae species identified to date are able to accumulate high concentrations of either lipids or starch, which are useful feedstock for various products (Hu et al. 2008). Furthermore, microalgae can accumulate various kinds of pigment, such as carotenoids, originating from endogenous photosynthesis-related pathways, which are usually absent in other microorganisms such as bacteria and yeast. More recently, microalgae have been considered as a promising host for recombinant protein production (Mayfield et al. 2003; Gimpel et al. 2015; Rasala and Mayfield 2015). Although recombinant protein production is currently dominated by Chinese hamster ovary (CHO) cells, microalgae offer various benefits during protein production. Microalgae are ideal for large-scale cultivation due to their rapid growth rates, short doubling time, and minimal nutritional requirements (Jahn et al. 2014). In addition, microalgae possess chaperones and protein disulfide isomerases in both the cytosol and plastid compartments and have glycosylation machinery, which can assist with the production of large and complex heterologous proteins (Kim and Mayfield 1997; Schroda 2004; Barrera and Mayfield 2013). Therefore, microalgae cultivations are generally more efficient compared to that of mammalian hosts or with other living organisms in terms of time and cost (Franklin and Mayfield 2004; Patil et al. 2008; Rawat et al. 2013).

In addition to microalgae-based production technologies, there has also been an increased interest in the genetic and cellular engineering of microalgae. To obtain desired properties in engineered microalgae, control of gene expression is important, and this is mostly regulated by promoters. To date, various constitutive promoters have been used for microalgal gene expression. For example, heterologous promoters such as cauliflower mosaic virus (CaMV) 35S promoter and nopaline synthase promoter (NOS) from *Agrobacterium* sp. have been widely used. Microalgae-derived promoters such as RBCS2, *β*-2-tubulin, and heat shock protein (*hsp*) 70A promoters from *Chlamydomonas reinhardtii* and violaxanthin/chlorophyll α-binding protein promoter (*vcp*) from *Nannochloropsis oculata* have also been popularly employed for various gene expression (Sun et al. 2005; Niu et al. 2011; Barrera and Mayfield 2013). However, the use of inducible promoters would be more ideal for the precise control of gene expression; hence, the attentions for the inducible promoters have been generated. Inducible promoters enable transgene expression to be induced at a desired time point, and the expression level can be easily controlled by varying the inducer concentration. Therefore, this method can be easily applied to various research and industrial fields. Despite its importance, few studies have investigated the inducible gene expression in microalgae; however, several inducible promoter systems have been reported (Poulsen and Kroger 2005; Li et al. 2008; Niu et al. 2011). Consequently, the development of efficient inducible promoters for microalgal species is challenging for both basic and applied phycology. Hence, in this study, we focused on the development of an inducible promoter system in microalgae.

Here, we designed an alcohol-inducible promoter system, originating from the filamentous fungus *Aspergillus nidulans*. This promoter system is composed of a regulatory protein, AlcR, and a promoter controlling *alc*A gene expression (P_*alc*A_) (Nikolaev et al. 2002). Using the AlcR-P_*alc*A_ system, inducible gene expression has previously been demonstrated in various plant species, such as *Arabidopsis thaliana*, *Populus* sp., and *Lycopersicon esculentum* (tomato) (Roslan et al. 2001; Garoosi et al. 2005; Filichkin et al. 2006). Hence, we expected that a similar result would be obtained upon using microalgae as a host. Indeed, when the promoter system was employed to express mCherry in the model strain *C. reinhardtii* CC-503, ethanol-dependent expression was observed. In addition, no uptake of ethanol was observed during cultivation, and no significant toxicity was associated with the inducer under the experimental conditions used.

## Materials and methods

### Plasmid construction

**Fig. 1:**
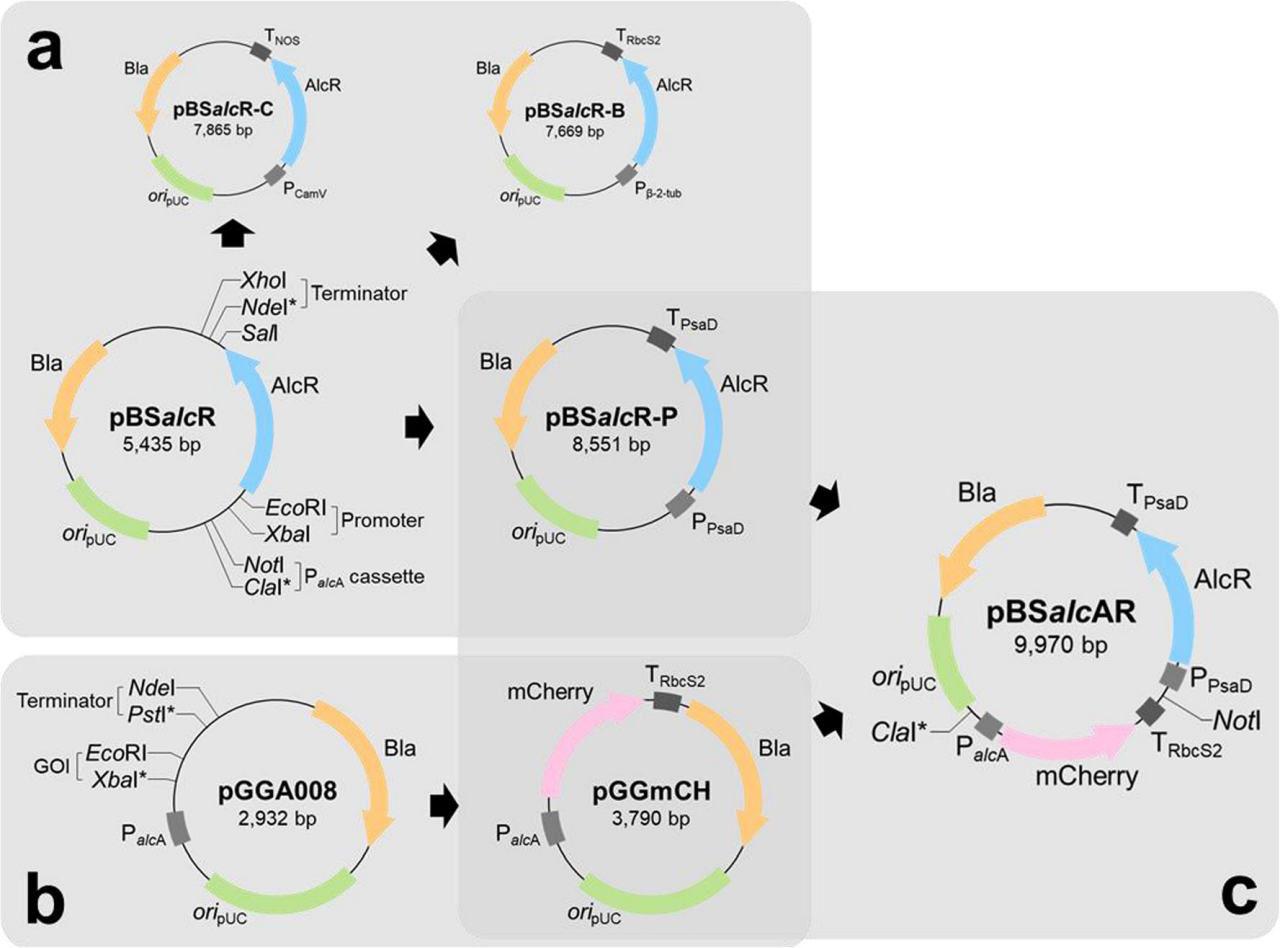
Schematic diagram of plasmid construction. **a** Plasmids related to expression of regulatory protein, AlcR. **b** Subcloning of codon-optimized mCherry-encoding gene into the P_*alc*A_ containing plasmid, pGGA008. **c** Construction of final expression plasmid, pBS*alc*AR. Asterisks (*) represent artificially generated restriction sites by site-directed mutagenesis. Abbreviations: Bla (beta-lactamase, ampicillin-resistance gene); *ori*_pUC_ (pUC19-derived origin of replication); P_camV_ (CamV 35S promoter); P_β-2-tub_ (*β*-2-tubulin promoter); P_PsaD_ (PsaD promoter); T_NOS_ (NOS terminator); T_RbcS2_ (RbcS2 terminator); T_PsaD_ (PsaD terminator); GOI (gene of interest)

All plasmids and oligonucleotide primers used in this study are listed in Table 1 and Supplementary Table S1, respectively. Gene accession numbers of promoters and terminators used in this study are also listed in Supplementary Table S2. For the heterologous expression of the regulatory protein, AlcR in *C. reinhardtii*, the gene was synthesized considering the codon preference of *C. reinhardtii* by a commercial service (Cosmo Genetech Inc., Seoul, Korea). The synthesized gene was cloned into pBluescript II SK(+) plasmid, containing EcoRI and SalI restriction sites; the recombinant plasmid was named pBS*alc*R0. Promoters and terminators were required for AlcR expression; there was no restriction site for the insertion of terminators in pBS*alc*R0, whereas promoters could be introduced using XbaI and EcoRI. Hence, an NdeI restriction site was generated downstream of the AlcR-encoding gene using the Muta-Direct site-directed mutagenesis kit (iNtRON, Seongnam, Korea) to introduce transcription terminators, yielding pBS*alc*R (Fig. 1a). Using this plasmid as a backbone, AlcR expression plasmids with different promoters and terminators were constructed. The PsaD promoter gene was amplified from pCR102 (Arabidopsis Biological Resource Center, Columbus, OH, USA) using the ‘Ppsad-F/R’ primer set, and the amplified DNA was digested and cloned into pBS*alc*R using XbaI and EcoRI restriction endonucleases. After PsaD promoter insertion, the PsaD terminator gene was amplified from pCR102 using the ‘Tpsad-F/R’ primer set, and the DNA fragment was digested and cloned into the PsaD promoter-inserted pBS*alc*R using NdeI and XhoI restriction endonucleases, to yield pBS*alc*R-P (Fig. 1a). To construct pBS*alc*R-B, the *β*-2-tubulin promoter gene was amplified from pJD100 (Chlamydomonas Resource Center, St. Paul, MN, USA) using the ‘Ptub-F/R’ primer set, and the RbcS2 terminator gene was amplified from pCR102 using the ‘Trbcs-F/R’ primer set, and then, each DNA fragment was cloned into the ‘XbaI-EcoRI’ and ‘NdeI-XhoI’ restriction sites, respectively. pBS*alc*R-C was similarly constructed using the CaMV 35S promoter and the NOS terminator from pCAMBIA1304 (Takara Bio, Japan). The primer sets ‘Pcamv-F/R’ and ‘Tnos-F/R’ were used for PCR and the same restriction sites were used to insert each gene.

**Table 1:** Plasmids used in this study

Plasmid	Relevant characteristics	Sources or Refs
pBluescript II SK (+)	Commercial gene cloning plasmid	Stratagene
pCR102	Deposited plasmid, containing PsaD promoter and terminator genes	ABRC
pCAMBIA1304	Commercial gene expression plasmid	Invitrogen
pGGA008	Deposited plasmid, containing *alc*A promoter sequence from *Aspergillus nidulans*	Addgene plasmid #48817
pBR_mCherry_Cr	Deposited plasmid, containing codon optimized mCherry-encoding gene	CRC
pBS*alc*R0	pBluescript II SK (+) containing codon optimized AlcR-encoding gene	Cosmo Genetech, Inc.
pBS*alc*R	pBSalcR0 containing artificially generated NdeI site	This study
pBS*alc*R-P	pBS*alc*R containing PsaD promoter and terminator	This study
pBS*alc*R-B	pBS*alc*R containing *β*-2-tubulin promoter and RbcS2 terminator	This study
pBS*alc*R-C	pBS*alc*R containing CamV 35S promoter and NOS terminator	This study
pGGmCH	pGGA008 containing mCherry-encoding gene from pBR_mCherry_Cr and RbcS2 terminator	This study
pBS*alc*AR	pBSalcR-P containing P_*alc*A_-mCherry-T_RbcS2_ cassette from pGGmCH	This study

To construct a model protein expression cassette, the gene encoding mCherry was subcloned into the pGGA008 plasmid (gift from Dr. Jan Lohmann; Addgene plasmid #48817; Fig. 1b) containing the P_*alc*A_ promoter. To subclone the mCherry gene, an XbaI restriction site was artificially generated (2550th TTTACA → TCTAGA) using the Muta-Direct site-directed mutagenesis kit (iNtRON). Then, the codon-optimized mCherry gene was amplified from pBR_mCherry_Cr (Chlamydomonas Resource Center) using the primer set ‘mCH-F/R’, and this gene fragment was digested and cloned into the XbaI site-generated pGGA008 using XbaI and EcoRI restriction endonucleases. For insertion of the transcription terminator, a PstI restriction site was generated by site-directed mutagenesis; the RbcS2 terminator was amplified from pCR102 (by using primer set of ‘Trbcs-2F/2R’) and subcloned to yield pGGmCH (Fig. 1b) using the PstI site and the inherent NdeI site. After the subcloning as described above, the P_*alc*A_-mCherry-T_RbcS2_ expression cassette was amplified by PCR using the primer set ‘mCHset-F/R’. Then, this fragment was digested and cloned into pBS*alc*R-P using ClaI and NotI restriction endonucleases, to yield pBS*alc*AR (Fig. 1c). Notably, pBS*alc*R-P does not contain a ClaI restriction site; therefore, site-directed mutagenesis was carried out to generate a ClaI site upstream of the NotI site, prior to gene cloning. Standard procedures were followed for all the experiments involving DNA manipulations and gene cloning (Green et al. 2012).

### Strains and cell cultivation

*Escherichia coli* DH5α was used in all the experiments for gene cloning, maintenance, and amplification. *E. coli* was cultured at 37 °C with shaking at 200 rpm. The culture medium was liquid LB broth (BD, USA), supplemented with an appropriate concentration of antibiotic (100 mg L^−1^ ampicillin), if necessary. The cell wall-deficient mutant strain, *C. reinhardtii* CC-503 cw92 mt+ was purchased from the Chlamydomonas Resource Center and used for all the protein expression experiments, unless otherwise stated. *Chlamydomonas reinhardtii* SM2 was also used as a positive control for mCherry expression; detailed information on the strains used in this study is provided in supplementary Table S3. Microalgae were cultivated in tris-acetate-phosphate (TAP) medium at 25 °C. During cell culture, light intensity was maintained (100 μmol photons m^−2^ s^−1^) using a light-equipped shaking incubator. For the cultivation of transgenic *C. reinhardtii*, an appropriate concentration of antibiotic (10 μg mL^−1^ paromomycin) was added. The number of cells in each culture was counted using a hemocytometer, C-Chip (iNCYTO, Cheonan, Korea), with 10 μL of each culture solution.

### Transformation of microalgae

*Chlamydomonas reinhardtii* were transformed using the Gene Pulser Xcell Electroporation System (Bio-Rad, USA). To enhance efficiency, the MAX Efficiency Transformation Reagent for Algae (Invitrogen, USA) was used to pretreat the cells (approximately 2 × 10^8^ cells mL^−1^) according to the manufacturer’s recommendation. Linearized plasmid (1 μg) was mixed with pretreated *C. reinhardtii* CC-503 cells in a 0.4-cm MicroPulser Electroporation Cuvette (Bio-Rad), and then, electroporation was carried out at 500 V, 50 μF, and 800 Ω. Immediately after electroporation, an appropriate amount of liquid TAP medium with 40 mM sucrose was added, and the cells were allowed to grow for 24 h. After 24-h recovery, the cells were collected by centrifugation at 2500×*g* for 10 min. The pellet was resuspended in a small volume of liquid TAP medium, and then, the suspended cells were gently spread on the TAP-agar plate with 10 μg mL^−1^ paromomycin.

### Reverse transcription quantitative polymerase chain reaction

Cells were fragmentized with liquid nitrogen using a mortar and pestle, and then RNA was extracted using the PureLink RNA mini kit (Invitrogen). Complementary DNA (cDNA) was synthesized using a reverse transcription kit (Promega, USA) by employing 1 μg of RNA extract as a template. Expression of the *alc*R gene in pBS*alc*R-P, pBS*alc*R-B, and pBS*alc*R-C was analyzed by RT-qPCR in a Peltier Thermal Cycler with a Chromo4 detector (Bio-Rad Technologies, USA). The 2^−ΔΔC^_T_ method was used to calculate fold changes in the gene expression levels. Gene expression was normalized based on the expression of the housekeeping gene, *Chlamydomonas* beta subunit-like polypeptide (CBLP). Wild-type *C. reinhardtii* CC-503 was used as a negative control. Expression of the target gene mCherry in pBS*alc*AR was confirmed by semi-quantitative PCR, followed by analysis of the PCR products on a 1% agarose gel. For both PCRs, 2 ng of cDNA was used as a template and the procedure was performed following the manufacturer’s recommendations.

### SDS-PAGE analysis and Western blotting

After cultivation, normalized number of cells was harvested; cells were disrupted, and protein was extracted using bead beating. After protein extraction, the total extract was boiled with SDS-PAGE sample buffer for 5 min (Green et al. 2012). The boiled samples were cooled and subjected to SDS-PAGE. After gel electrophoresis, the proteins were transferred to polyvinylidene fluoride (PVDF; Bio-Rad) membranes, which were then blocked by 5% (*w*/*v*) skim milk solution in Tris-buffered saline containing Tween-20 (TBST) for 1 h. After blocking, the membranes were washed four times with TBST. The membranes were incubated in 1:2000 diluted mouse anti-mCherry monoclonal antibody [1C51] (Abcam, Cambridge, UK) in blocking solution at 25 °C. After 1 h, the primary antibody solution was discarded, and the membranes were incubated in 1:10,000 diluted HRP-conjugated goat anti-mouse IgG H&L antibody (Abcam) for 1 h. The membranes were washed four times with TBST for 10 min, and then subjected to chemiluminescent analysis. The bands were visualized using ECL Western blotting substrate (Promega) and detected using a Chemiluminescence Imaging System (Atto Korea, Daejeon, Korea).

## Results

### Ethanol tolerance of *C. reinhardtii* CC-503

**Fig. 2:**
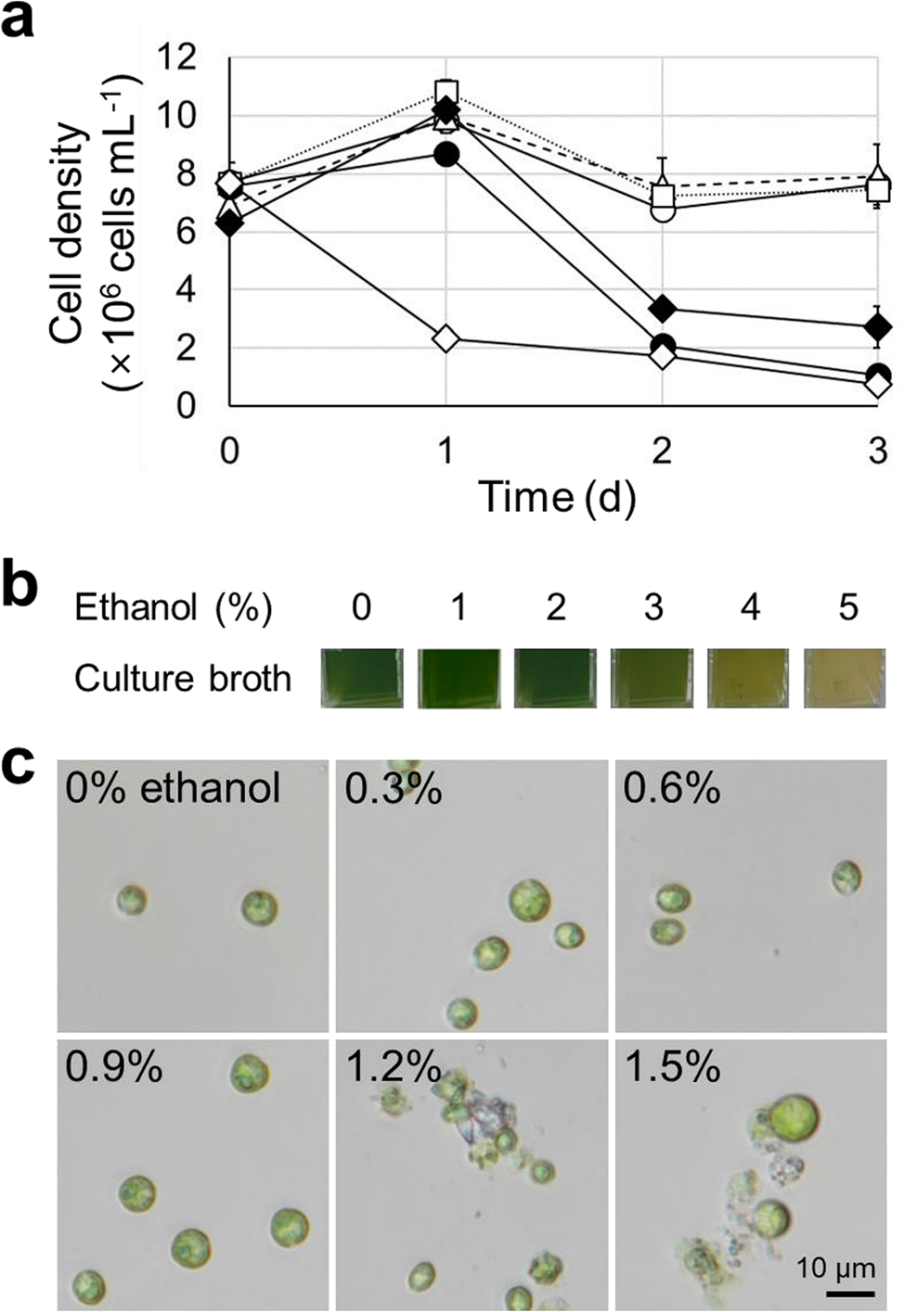
Ethanol tolerance of *Chlamydomoas reinhardtii* CC-503. **a** Growth profile of *C. reinhardtii* CC-503 under the different concentrations of ethanol. Open circle (○), open triangle (△; dash line), open square (□; dot line), closed diamond (◆), closed circle (●), and open diamond (◇) represent 0, 1, 2, 3, 4, and 5% of ethanol, respectively. Error bars represent the standard deviation of the biological triplication. **b** The color of culture broth after 3-day cultivation. Each number of picture represents initial ethanol concentration of culture medium. **c** Microscopic image of *C. reinhardtii* CC-503 after 2-day cultivation under the different concentrations of ethanol. Ethanol concentrations are denoted at the left top of each image

Ethanol is required to activate AlcR in order to induce target genes under the control of the *alc*A promoter (P_*alc*A_) (Nikolaev et al. 2002). However, significant concentrations of alcohol, specifically ethanol used in this study, are toxic to a broad range of microorganisms (Zingaro and Papoutsakis 2013; Haft et al. 2014). Hence, the tolerance of *C. reinhardtii* CC-503 to alcohol was examined to assess the possibility of using ethanol as an inducer. Three days after cell inoculation, *C. reinhardtii* cultures in the late-exponential phase (approximately 7 × 10^6^ cells mL^−1^) were exposed to different concentrations of ethanol (1, 2, 3, 4, and 5% as the final concentrations), and the cell densities were subsequently monitored during further cultivation. In the absence of ethanol (0%), cell density remained constant during the 3-day monitoring period. In addition, cells exposed to 1 and 2% ethanol exhibited a similar growth profile compared to the ethanol-free sample (Fig. 2a). However, higher concentrations of ethanol led to severe defects in cell viability. Cells were significantly damaged immediately after exposure to 5% ethanol, such that the cell density decreased markedly. With the addition of 3 and 4% ethanol, severe growth retardation was also observed; however, this occurred 1 day after the treatment (Fig. 2a). During the cultivation, the color of culture broth was also paled out proportional to the ethanol concentration. After 3-day cultivation, samples treated with more than 3% ethanol exhibited sequentially whitened colors (Fig. 2b). To further evaluate cell viability, the cells were exposed to relatively low concentrations of ethanol (0.3, 0.6, 0.9, 1.2, and 1.5%) for 2 days, followed by microscopic analysis. When the cells were exposed to ethanol concentrations lower than 1% (0.3, 0.6, and 0.9%), almost all cells exhibited intact morphology without significant defects (Fig. 2c). In contrast, some of the cells exposed to 1.2 and 1.5% ethanol appeared to be damaged (Fig. 2c). Based on these results, it was deduced that the cells grown with 2% ethanol may contain high portions of damaged cells, in spite of the growth was not retarded, since it was measured by counting the cells through hemocytometer. Consequently, we used ethanol at concentrations lower than 1% for further experiments, although cell growth was not significantly affected under 2% ethanol.

### Evaluation of ethanol consumption by *C. reinhardtii* CC-503

**Fig. 3:**
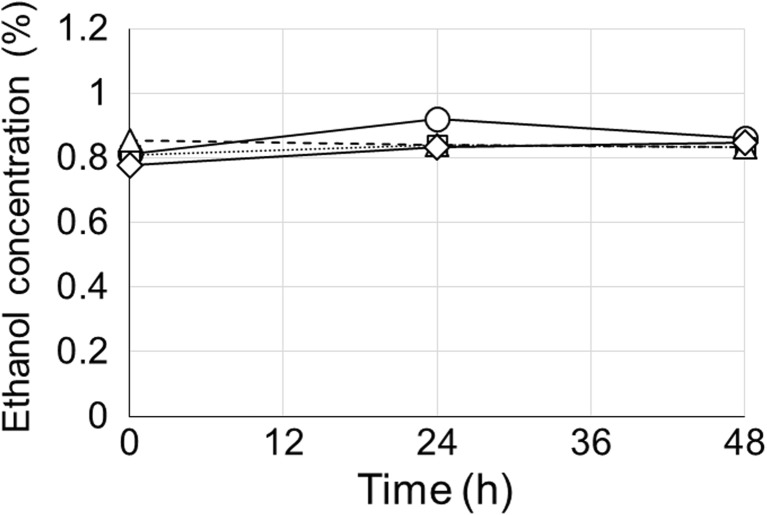
Ethanol consumption profile during the 2-day cultivation of the different *C. reinhardtii* strains. Circle (○), triangle (△; dash line), diamond (◇), and square (□; dot line) represent CC-503 WT, CC-503 mut1, CC-503 mut2, and Sta6, respectively. Detailed information of each strain is described in supplementary Table S2

In most metabolite-inducible promoter systems inducer consumption is one of the biggest obstacles preventing effective utilization (Hsieh and Da Silva 2000). For instance, the *E. coli* lac promoter has been widely used with isopropyl β-D-1-thiogalactopyranoside (IPTG; non-metabolizable allolactose analog) induction to avoid lactose consumption. In order to utilize the ethanol-inducible promoter, P_*alc*A_, we investigated the rate of ethanol consumption by four different *C. reinhardtii* strains (CC-503, CC-503 mut1, CC-503 mut2, and sta6) in 1% ethanol. Following ethanol treatment during the late exponential phase of cultivation, the ethanol concentration in the culture supernatant at each time point was analyzed by high-performance liquid chromatography (HPLC). In the case of CC-503, there was no significant change in the concentration of ethanol during the 2-day cultivation period (Fig. 3). Similarly, there was no change in the ethanol concentration in the other three strains during the same period (Fig. 3).

### Expression of regulatory protein (AlcR) in *C. reinhardtii*

**Fig. 4:**
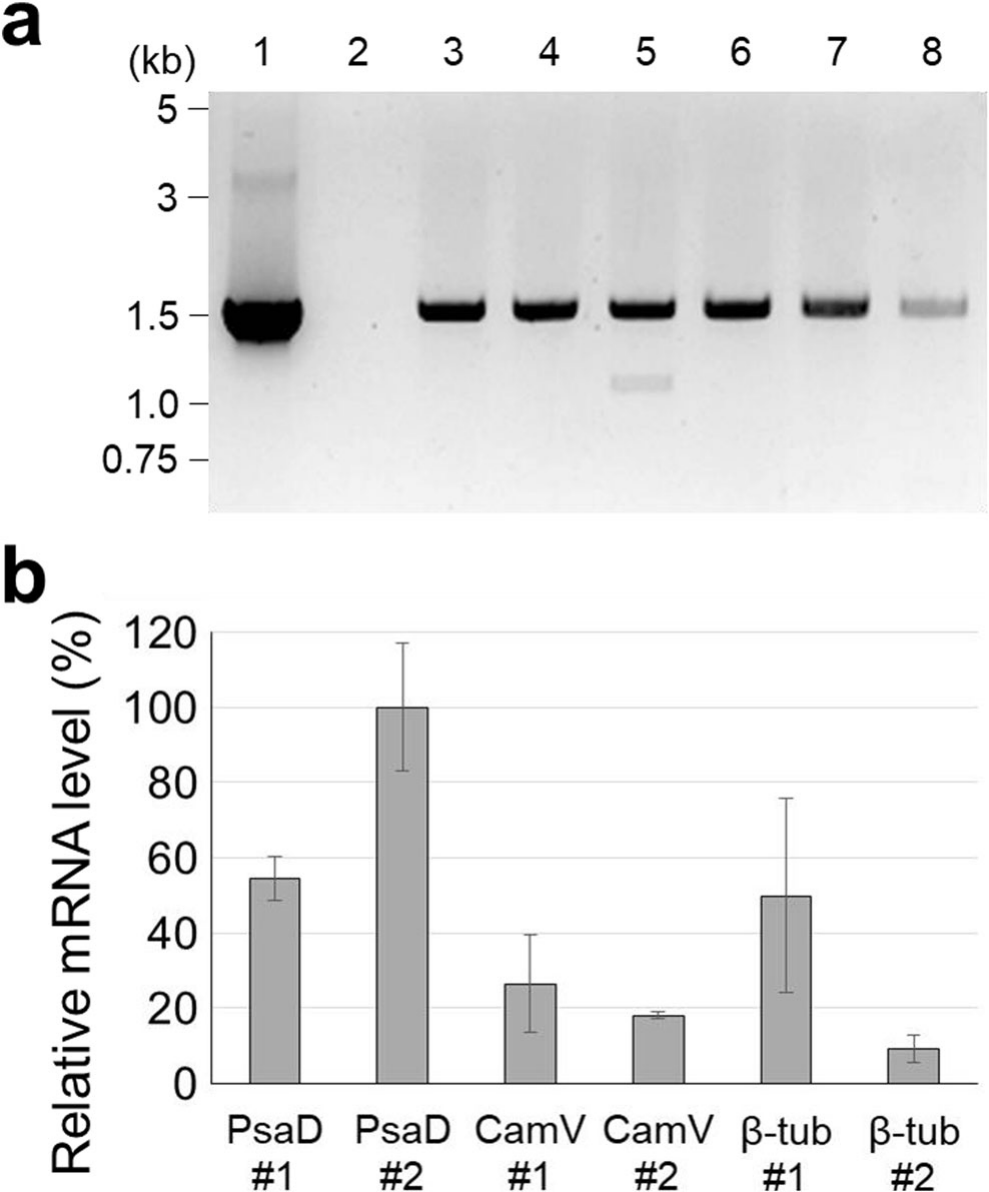
Expression of regulatory protein, AlcR with different combinations of promoters and terminators. **a** Transformation confirmation of AlcR-encoding gene by using PCR. Genomic DNA extract of each transformant was used as a template. Lanes 1 and 2 represent positive (pBS*alc*R as template) and negative (gDNA from WT cells) controls, and lanes 3, 4, 5, 6, 7, and 8 represent PCR reactant when using gDNA from PsaD #1, PsaD #2, CamV #1, CamV #2, β-tub #1, and β-tub #2 as templates, respectively. **b** Relative expression level of AlcR from the different kinds of transformants. The expression level was measured by RT-qPCR

The ethanol-inducible promoter, P_*alc*A_, is unable to respond to ethanol directly; however, the ethanol-activated regulatory protein (AlcR) is able to induce the expression. For this reason, AlcR must be expressed at a significant level in the target strain. To optimize the level of AlcR expression, three plasmids (pBS*alc*R-P, pBS*alc*R-C, and pBS*alc*R*-*B) with different promoter and terminator combinations were prepared. *Chlamydomonas reinhardtii* CC-503 was transformed using each plasmid, resulting in three different recombinant strains with distinct AlcR expression cassettes. Transformations were successfully carried out with the efficiency range of 10^2^ to 10^4^ cfu μg^−1^. The transgene was maintained well in the subcultures during the rest of experiment. After the transformation, two colonies for each strain were randomly selected and gDNA was extracted for use in PCR to confirm gene integration. A 1.5 kb band was detected in all the samples, and the size of the band was consistent with that of the positive control (Fig. 4a). In addition, the level of *alc*R gene transcription was determined by RT-qPCR to evaluate the expression of each transformant. Both transformants harboring pBS*alc*R-P (PsaD #1 and #2) exhibited high levels of *alc*R transcripts. When the relative mRNA expression was determined by setting the highest value at 100%, each transformant expressed 59.6 and 100% mRNA, respectively (Fig. 4b). In contrast, low levels of AlcR expression were found in the cells transformed with pBS*alc*R-C or pBS*alc*R-B. Transformants harboring pBS*alc*R-C (CamV #1 and #2) that contained the CaMV 35S promoter expressed 28.3 and 18.2% of AlcR transcripts, whereas 26.4 and 4.1% mRNA was expressed from β-tub #1 and #2 (*β*-2-tubulin promoter) transgenic algal strains (Fig. 4b). Based on this, we concluded that the PsaD promoter is more appropriate to drive AlcR expression, and thus, the expression cassette with this promoter was employed for further studies.

### Expression of mCherry under the control of an ethanol-inducible promoter

**Fig. 5:**
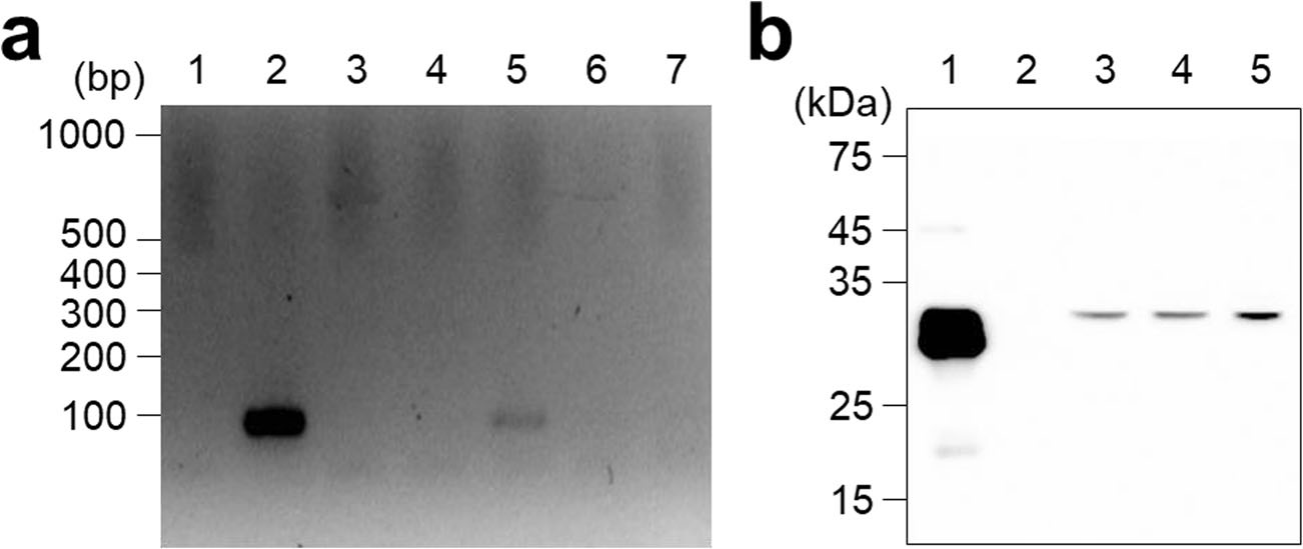
Ethanol-dependent expression of mCherry. **a** Semi-quantitative PCR of mCherry transcript. Lanes 1, 2, 3, 4, 5, 6, and 7 represent when using water, pBS*alc*AR, CC-503 WT, CC-503 mut1, P18, P26, and P28 as templates, respectively. **b** Western blot analysis of mCherry expression from P18 transformant under the different concentrations of ethanol. Lanes 1, 2, 3, 4, and 5 represent positive control, negative control, 0%, 0.5%, and 1% ethanol, respectively

To demonstrate the ethanol-inducible gene expression with P_*alc*A_, the mCherry expression plasmid (pBS*alc*AR), which contains a gene encoding mCherry under the control of P_*alc*A_ and the expression cassette of AlcR from the pBS*alcR*-P, was constructed. *Chlamydomonas reinhardtii* CC-503 was transformed with pBS*alc*AR, and then, 60 colonies were randomly selected and cultured in liquid TAP medium. Ethanol induction (1%) was performed when the cells reached the late-exponential phase, followed by 2-day post-induction cultivation for subsequent analysis. After cultivation, the fluorescence signal from each clone was first measured using a microplate fluorescence reader to screen out the cells expressing mCherry in the presence of 1% ethanol. Some clones exhibited increased fluorescence (data not shown), and the three clones (P18, P26, and P28) with the highest fluorescence were selected and subjected to semi-quantitative PCR (semi-qPCR). After mRNA preparation followed by cDNA synthesis from the three clones, semi-qPCR was carried out using the cDNA samples as templates. As a result, P18 showed a 100 bp band, consistent with the positive control (Fig. 5a). In contrast, no band was detected in P26 and P28 samples, consistent with the negative controls (Fig. 5a). Based on this, SDS-PAGE followed by Western blot analysis was carried out with the P18 transformant to examine the expression of mCherry. To verify whether the expression was dependent on the inducer treatment, P18 cells were cultivated and exposed to different concentrations of ethanol (0, 0.5, and 1%), and then, each sample was subjected to Western blot analysis. Although ethanol was not used to induce gene expression, leaky expression was observed in the sample without ethanol (Fig. 5b). When 0.5% ethanol was added, a band of the same size as that of the positive control was also detected; however, the intensity was similar to that of 0% ethanol sample, despite the addition of ethanol (Fig. 5b). In contrast, a thicker band was detected under 1% ethanol condition, indicating that ethanol could induce the expression of the mCherry gene under the control of P_*alc*A_. To quantify the induced expression, densitometric analysis was performed with ImageJ software, and a 1.74-fold increase in the expression was observed with 1% ethanol, whereas a 1.12-fold increase in the expression was observed with 0.5% ethanol.

## Discussion

Gene manipulation is still challenging in most microalgal species. There is an increasing need to perform genetic and cellular engineering in microalgae, and the development of functional promoters is important for this purpose. For the recombinant gene expression in microalgae, a number of different promoters have been developed; however, most are constitutive promoters, and of which control is quite limited. Based on the successful use of inducible promoters in microbial or animal hosts, similar efforts have been made to develop a controllable expression system in microalgae, with some progress. The *Phaeodactylum tricornutum*-derived nitrate reductase (NR) gene promoter has been most commonly used for the heterologous expression of proteins. This promoter can be induced by nitrate, and subsequently drives the expression of target proteins, such as enhanced green fluorescence protein (EGFP) and chloramphenicol acetyltransferase (CAT) (Sun et al. 2006; Li et al. 2007; Li et al. 2008; Niu et al. 2011). In addition, several stress-inducible promoters have been developed and used to produce valuable products. Iwai and colleagues introduced the phosphorous starvation-inducible promoter, *SQD2*, to enhance the TAG content of *C. reinhardtii* (Iwai et al. 2014). They also reported enhanced oil synthesis of *Nannochloropsis* using the same promoter (Iwai et al. 2015). Copper-, cadmium-, or iron-stress-inducible promoters were also studied and used to produce valuable products such as hydrogen (Rubinelli et al. 2002; Surzycki et al. 2007). Although progress has been made, it has mostly focused on the endogenous inducible promoters; therefore, the expression might be influenced by the complicated endogenous pathways of the host organism rather than being affected by the intended inducer. In addition, stress-inducible promoters were successfully utilized to enhance the production of specific products in some previous studies; however, their application is limited to the target host and product. In contrast, the AlcR-P_*alc*A_ system is composed of only two molecular components. Therefore, this compact system is expected to be used for a broad range of hosts or products (Filichkin et al. 2006). Furthermore, the AlcR-P_*alc*A_ system originated from the fungus *Aspergillus nidulans*; hence, the orthogonality of the expression control is more ensured in comparison to microalgae-based endogenous promoters. In addition, since alcohol is used to induce the AlcR-P_*alc*A_ system and is a common metabolite in various living organisms, it is relatively free from safety concerns in industrial applications. Despite the theoretical orthogonality, leaky expression, which is a common problem with inducible promoters in various hosts (Celesnik et al. 2016; Huang et al. 2015), was observed in Western blot analysis. Plus, the final level of expression observed in response to 1% ethanol was not satisfactory. However, there was a clear relationship between the presence of ethanol and the expression level, and there was no significant toxicity or ethanol uptake. Therefore, the AlcR-P_*alc*A_ system represents a suitable choice for the controllable expression of proteins in microalgae. Furthermore, reducing the leaky expression and improving target gene expression are additional challenges; thus, further research on these issues is required to improve microalgae-based gene expression technology.

## Electronic supplementary material

ESM 1 (DOCX 18 kb)
