# Supplementary material for: Development of an alcohol-inducible gene expression system for recombinant protein expression in Chlamydomonas reinhardtii
Source: J Appl Phycol. 2018 Apr 24;30(4):2297–304. doi: 10.1007/s10811-018-1480-8 (PMC6096782; doi:10.1007/s10811-018-1480-8)
Supplement: Supplementary file 1 — (DOCX 18 kb) [file 10811_2018_1480_MOESM1_ESM.docx]

[Supplementary Materials]

**Development of an alcohol-inducible gene expression system for recombinant protein expression in *Chlamydomonas reinhardtii***

Sujin Lee ∙ Yong Jae Lee ∙ Saehae Choi ∙ Su-Bin Park ∙ Quynh-Giao Tran ∙ Jina Heo ∙ Hee-Sik Kim

**Table S1.** Oligonucleotide primers used in this study

| Name | Sequences (5′ → 3′) |
| --- | --- |
| Ppasd-F | CGATCTAGACTCGAAATTAACCCTCACTAAAGGGAA |
| Ppasd-R | CGAGAATTCCAGGCGGTCCAATCTGAATTGGGCTGC |
| Tpasd-F | CGACATATGCTCGGAGGCTTTCGCGCT |
| Tpasd-R | CGACTCGAGGGGGAGACAGCGTGACTGTG |
| Ptub-F | CGATCTAGATGCATGCAACACCGAT |
| Ptub-R | GCTGAATTCTTTGCGGGTTGTGACTG |
| Trbcs-F | CGACATATGTAAGGATCCCCGCTCCGGTGTAAATGG |
| Trbcs-R | CGACTCGAGGCATGGAGAAAGAGGCC |
| Pcamv-F | CGATCTAGACAGAAGACCAAAGGGCAA |
| Pcamv-R | GCTGAATTCAGAGTCCCCCGTGTTC |
| Tnos-F | CGACATATGGATCGTTCAAACATTTGGCAATAAAG |
| Tnos-R | CGACTCGAGGATCTAGTAACATAGATGACACCGC |
| mCH-F | GCATCTCTAGATTACTTGTACAGCTCGTCCAT |
| mCH-R | GCTGAATTCATGGTGTCCAAGGGCGA |
| Trbcs-2F | CGACTGCAGTAAGGATCCCCGCTCCGGTGTAAATGG |
| Trbcs-2R | CGACATATGGCATGGAGAAAGAGGCC |
| mCHset-F | TTTCACACGAGCTATTTAGGTGACACTATAGAAGTGAAGCTTGGTCT |
| mCHset-R | CTTAACTATGCGGCATCAGAGCAGATTGTACTGAGAGTGC |
| mCherry_ read_2F | ACATCAAGCTGGACATCACC |
| mCherry_ read_2R | CTTGTACAGCTCGTCCATGC |

**Table S2.** Promoters and terminators used in this study

| Gene | Description | GeneBank Accession No. | Length in use |
| --- | --- | --- | --- |
| P_PsaD_ | *Chlamydomonas reinhardtii* photosystem I subunit (PsaD) gene promoter | AF335592 | 815 bp |
| P_CamV_ | Cauliflower mosaic virus 35S promoter | V00140 | 346 bp |
| P_β-2-tub_ | *Chlamydomonas reinhardtii* beta-2 tubulin gene promoter | K01809 | 250 bp |
| T_PsaD_ | *Chlamydomonas reinhardtii* photosystem I subunit (PsaD) gene terminator | AF335592 | 543 bp |
| T_NOS_ | *Agrobacterium tumefaciens* nopaline synthase (NOS) gene terminator | AJ007623 | 253 bp |
| T_RbcS2_ | *Chlamydomonas reinhardtii* ribulose bisphosphate carboxylase/oxygenase small subunit (RbcS2) gene terminator | X04472 | 211 bp |

**Table S3.** Strains used in this study

| Strains | Relevant characteristics | Sources or References |
| --- | --- | --- |
| *Escherichia coli* DH5α | Gene cloning strain | RBC |
| *C. reinhardtii* CC-503 | Cell wall-deficient mutant of *C. reinhardtii* | Chlamydomonas Resource Center |
| *C. reinhardtii* sta6 | Starchless mutant of *C. reinhardtii* | Chlamydomonas Resource Center |
| CC-503 mut1 | Unidentified transformant of *C. reinhardtii* CC-503 #1 | This study |
| CC-503 mut2 | Unidentified transformant of *C. reinhardtii* CC-503 #2 | This study |
| *C. reinhardtii* SM2 | Transgenic *C. reinhardtii* harboring mCherry-encoding plasmid | Laboratory stock |
| PsaD #1 | Transgenic *C. reinhardtii* CC-503 harboring pBS*alc*R-P #1 | This study |
| PsaD #2 | Transgenic *C. reinhardtii* CC-503 harboring pBS*alc*R-P #2 | This study |
| CamV #1 | Transgenic *C. reinhardtii* CC-503 harboring pBS*alc*R-C #1 | This study |
| CamV #2 | Transgenic *C. reinhardtii* CC-503 harboring pBS*alc*R-C #2 | This study |
| β-tub #1 | Transgenic *C. reinhardtii* CC-503 harboring pBS*alc*R-B #1 | This study |
| β-tub #2 | Transgenic *C. reinhardtii* CC-503 harboring pBS*alc*R-B #2 | This study |
| P18 | Selected transgenic *C. reinhardtii* CC-503 harboring pBS*alc*AR #18 | This study |
| P26 | Selected transgenic *C. reinhardtii* CC-503 harboring pBS*alc*AR #26 | This study |
| P28 | Selected transgenic *C. reinhardtii* CC-503 harboring pBS*alc*AR #28 | This study |
